# Advanced remote focus control in multicore meta-fibers through 3D nanoprinted phase-only holograms

**DOI:** 10.1038/s41467-024-55805-7

**Published:** 2025-01-08

**Authors:** Mohammadhossein Khosravi, Torsten Wieduwilt, Matthias Zeisberger, Adrian Lorenz, Markus A. Schmidt

**Affiliations:** 1 Leibniz Institute of Photonic Technology, Jena, Germany; 2 Abbe Center of Photonics and Faculty of Physics, FSU Jena, Jena, Germany; 3 Otto Schott Institute of Material Research, FSU Jena, Jena, Germany

**Keywords:** Fibre optics and optical communications, Micro-optics, Integrated optics

## Abstract

In this study, we present an unexplored approach for remote focus manipulation using 3D nanoprinted holograms integrated on the end face of multi-core single-mode fibers. This innovative method enables precise focus control within a monolithic metafiber device by allowing light coupled into any of the 37 cores to be precisely focused at predefined locations. Our approach demonstrates significant advances over conventional lenses and offers unique functionalities through computationally designed holograms. This research marks the first successful use of multi-core fibers for remote focus control via 3D nanoprinting, achieving crosstalk-free operation at visible wavelengths. Key findings include strong agreement between design, simulation, and experimental results, highlighting the potential of this technology to improve applications in fields such as biological optics, laser micromachining, telecommunications, and laser surgery. This work opens new avenues for the development of advanced optical systems with superior focus control capabilities.

## Abstract (web summary)

Researchers demonstrate remote focus control in multicore fibers via 3D nanoprinted holograms. This enables precise, crosstalk-free focusing, advancing optical, biophotonics, and telecommunications applications.

## Introduction

Light focusing at specific locations is fundamental for nearly all optical systems and is critical in many scientific and application areas, including fields as diverse as telecommunications (e.g., wavelength multiplexing^1^), medicine (e.g., laser ablation^2^), biophotonics (e.g., optogenetics^3,4^), and materials science (e.g., laser micromachining^5^).

Many applications require remote and ideally reconfigurable focusing of light, which is generally difficult to achieve with bulk optics. With the ability to deliver light over long distances while maintaining high transmission quality, optical fibers have become indispensable in modern photonics due to excellent light transportation capabilities. Many practical fibers consist of a high refractive index core surrounded by a low refractive index glass cladding (usually silica), forming solid glass fibers with a step-index type profile. These fibers have revolutionized photonics in various fields such as telecommunications^6^, quantum technologies^7^, and sensing^8^, thus suggesting a promising solution for remote light focusing when integrated with optical focusing capabilities.

In the context of controlling the flow of light, a key area of photonics research is dielectric nanostructures for complex beam manipulation^9^, primarily focusing on metasurfaces (MSs) and phase-only holograms. MSs, which consist of arrays of subwavelength elements with engineered phase and amplitude properties, enable multimodal beam shaping^10,11^, including for instance orbital angular momentum beam generation^12^. However, their fabrication, especially when coupled to optical fibers, presents challenges. In contrast, phase-only holograms offer a more efficient solution for phase and intensity modulation, with simpler designs that enhance mechanical stability, reduce cost, and improve diffraction efficiency. They have found successful applications in fields such as optical tweezers^13^, augmented reality^14^, and metrology^15^.

Consequently, the transfer of planar nanophotonic effects to optical fiber end surfaces represents a promising direction for both fundamental research and practical applications and marks a significant trend toward the development of so-called meta-fibers. Unlike traditional lithographic methods, 3D nanoprinting is well suited to the geometry of fibers and facilitates the manipulation of the height of individual elements^16,17^. Notable on-fiber research examples include phase-only holograms for optical tweezers^18^, phase-controlled multifocal arrays^19^, MS-based structures for achromatic light focusing^20^ and complex beam shaping^21^ and gratings-type structures for improved light incoupling^22,23^.

One drawback of all mentioned on-fiber devices is that the generated focus remains static, while many applications demand real-time variable focus manipulation such as optical trapping and optical tweezers^24^, multiplexing in telecommunications^25^, or laser-based 3D nanoprinting^26^. Here, reconfigurable optics is a promising field with the potential to transform technologies by enabling dynamic control of optical properties, increasing versatility and efficiency in applications such as imaging, sensing, and telecommunications^27^. From a technological standpoint, developing an integrated, fiber-coupled platform that reduces dependence on bulk optical components would decrease the system’s geometric footprint and cost, creating new opportunities in reconfigurable optics.

Multicore fibers with single-mode cores (SM-MCFs) are a topical research focus in fiber optics, overcoming key limitations of traditional fibers. One class of SM-MCFs is specifically designed to suppress modal crosstalk by confining power to individual cores^28^. This improves system performance in several applications, including power scaling in fiber lasers^29,30^, increasing data rates in telecommunications^31^, imaging complex tissues^32^, beam shaping^33^, and designing highly efficient sensors^34,35^. Here, SM-MCFs provide a compelling platform for complex beam generation, as demonstrated by the coherent combination of multiple phase- and amplitude-tuned output beams from MCF amplifiers^33^ and high-power laser applications^36^, as well as for studying nonlinear pulse dynamics within MCF systems^37,38^. Consequently, interfacing SM-MCFs with phase-only holograms is expected to boost the performance of optical fibers and open up untapped applications, especially in tunable remote light focusing and fields related.

**Fig. 1:**
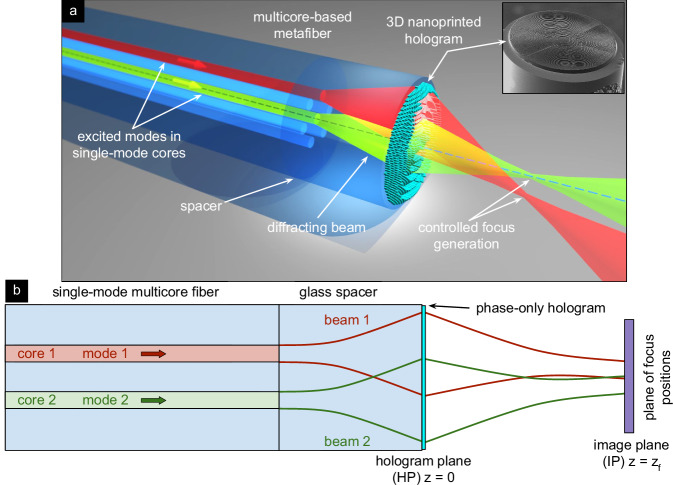
Tunable focus manipulation using multicore-fiber interfaced phase-only hologram. **a** Artistic view. To illustrate the functional principle, the modes of two cores are excited, resulting in two foci at different locations in the image plane (dashed blue line: central fiber axis). top right inset: example of a realized hologram on the fiber. **b** Sketch of the concept, including SM-MCF and glass spacer (light blue), phase-only hologram (cyan), image plane (purple). The beams and the modes in the correlated cores are represented by the green and red colors.

In this study, we address the aforementioned aspect by introducing and experimentally validating an unexplored approach for remote focus manipulation by integrating computationally designed 3D nanoprinted polymeric holograms onto the end face of single-mode multicore fibers (Fig. 1). This technique allows light coupled into any of the 37 individual cores to be precisely focused at predefined locations within the image plane, enabling sophisticated remote light control within a monolithic metafiber device. The method uses computational design and advanced 3D nanoprinting technology to create phase-only holograms that enable remote-focusing configurations with capabilities beyond those of conventional lenses. This work represents the first demonstration of the use of multi-core fibers for remote focus control via nanostructured holograms designed for crosstalk-free operation at visible wavelengths.

## Results

The concept of remote focus manipulation involves the use of SM-MCFs in combination with computer-designed, nanostructured phase-only holograms. This arrangement links each fundamental mode in the different cores with a focus position in the image plane (IP, located at *z* = *f*, *f*: focal length). This design allows dynamic control of the focus position in the IP by selectively exciting a different core. The SM-MCFs contain *N*_c_ = 37 cores and are designed to have no intermodal crosstalk in order to ensure a correlation between the light entering and exiting each core. To increase the efficiency of the nanostructured hologram, and to fully utilize the hologram area, a coreless fiber section is added between the fiber surface and the hologram, allowing the beam from each core to expand via diffraction.

The design of the SM-MCFs used in this study effectively eliminates modal crosstalk across all cores (operation wavelength *λ*_0_ = 637 nm), which is achieved by careful selection of the inter-core pitch (*Λ*_des_ = 15 μm). Uniformity of all cores (i.e., modes) was confirmed by determining the numerical aperture (*NA*_MCF_ of selected cores through measuring the beam profile at various distances from the fiber surface and fitting the data to a Gaussian Beam (GB) model. The results show near-perfect uniformity, with numerical aperture values consistently matching (*NA*_MCF_ = 0.08, see example curves in supplementary note 1). Note that a detailed study of the technical details of the SM-SMCF used is in preparation for a separate publication.

The hexagonal arrangement of the 37 cores is key for the concept presented here, as it defines the coordinates of the center of each GB in the hologram plane (HP, ${{\bf{r}}}_{{\rm{j}}}=\left({x}_{{\rm{j}}},{y}_{{\rm{j}}},z=0\right),\,j=1\ldots {N}_{{\rm{hol}}},\,{N}_{{\rm{hol}}}$: number of cores for the hologram design). Thus, the positions of an individual core and of a specified focus location in the IP (${{\bf{R}}}_{{\rm{j}}}=\left({X}_{{\rm{j}}},{Y}_{{\rm{j}}},z=f\right)$) are correlated by the positions of the GB in the HP (**r**_j_).

The phase-only holograms used in this study, represented by the phase distribution Φ(*x*, *y*), consist of nanoprinted dielectric elements with sub-wavelength dimensions that precisely manipulate the phase of passing waves. The height of these elements is calculated using:

1 $$h(x,y)=\Phi (x,y)\cdot {\lambda }_{0}/(2\pi (n-1))$$

where *n* is the refractive index of the polymer at *λ*_0_^18^. These holograms were designed using iterative phase retrieval based on the Gerchberg-Saxton Algorithm (GSA, described in supplementary note 2) in combination with the angular spectrum method (ASM, described in supplementary note 3) to calculate forward and backward beam propagation^39^. This combination results in a computational design scheme that is not limited to Fraunhofer or Fresnel domains.

The design for the phase-only hologram relies on considering the six outermost cores and the central core (*N*_h_ = 7, inter-core spacing *Λ*_des_ = 45 μm), together with seven selected focal positions in the IP. These cores were chosen as they have the maximum possible spacing between the cores and thus the GBs in the HP. The design procedure involves the following three steps:

- Step 1: The individual holograms of the respective pairs of GBs and focus positions (i.e., **r**_j_ and **R**_j_) were calculated using the ASM-based GSA, resulting in seven individual phase holograms defined by:
  2 $${\Phi }_{\,\text{j}}^{{\rm{HP}}}={\Phi }_{{\rm{j}}}^{\text{HP}\,}(x,y,z=0)$$
- Step 2: The phase distribution of the final hologram is determined by superimposing the electric fields in the HP. Each field is individually defined by:
  3 $${E}_{\,\text{j}}^{{\rm{HP}}}=\sqrt{{I}_{{\rm{j}}}^{\text{GB}\,}}\cdot \exp \left(i\cdot {\Phi }_{\,\text{j}}^{\text{HP}\,}\right)$$
  with the intensity distribution of the GB in the HP ${I}_{\,\text{j}}^{\text{GB}\,}$, leading to the total field:
  4 $${E}_{\,\text{tot}}^{{\rm{HP}}}=\mathop{\sum }\limits_{\text{j}=1}^{{\text{N}}_{{\rm{hol}}}}{E}_{{\rm{j}}}^{\text{HP}\,}=A\cdot \exp \left(i\cdot {\Phi }_{{\rm{hol}}}\right)$$
  with the amplitude *A* and the phase hologram Φ_h_. Note that the superposition of electric fields, rather than just phases, is used here, following standard holography practices commonly used in holography-related applications^40^.
- Step 3: The intrinsic curvature of the wavefronts of the GBs in the HP is compensated for by using a heuristically found procedure that relies on superimposing all GBs,
  5 $${E}_{\,\text{tot}}^{{\rm{GB}}}(z=0)=\mathop{\sum }\limits_{\text{j}=1}^{{\text{N}}_{{\rm{hol}}}}{E}_{{\rm{j}}}^{{\rm{GB}}}(z=0)={E}_{\text{tot},0}^{\text{GB}\,}\cdot \exp \left(i\cdot {\Phi }_{\,\text{tot}}^{\text{GB}\,}\right)$$
  (total amplitude and phase ${E}_{\,\text{tot},0}^{\text{GB}\,}$ and ${\Phi }_{\,\text{tot}}^{\text{GB}\,}$) and subtracting the resulting phase from the previously determined phase distribution:
  6 $$\Phi={\Phi }_{{\rm{hol}}}-{\Phi }_{{\rm{tot}}}^{\text{GB}\,}$$
  This phase distribution incorporates the contributions from all individual holograms and is compensated for the mean phase of all GBs in the HP.

### Implementation and experiments

The MCF was fabricated by the stack-and-draw method (outer diameter: OD = 200 μm) and contains 37 GeO_2_-doped cores (core diameter: 4.4 *μ*m, *NA*_MCF_ = 0.08, single-mode at *λ*_0_ = 637 nm).

To ensure free diffraction between fiber surface and meta-lens, a coreless glass fiber-type spacer (homemade, F300 silica glass, diameter 200 μm was spliced onto the MCF (length 1 m). To ensure that the resulting beam has a width of 60 μm in the HP and thus extends over the entire hologram area, the spacer was cut to a length of about *L*_s_ = 565 μm.

3D nanoprinting of on-fiber holograms was performed using femtosecond direct laser writing (fs-DLW) via two-photon absorption with a commercial photolithography system (Photonic Professional GT, Nanoscribe GmbH, details in Methods). This technique offers several key advantages, including high spatial resolution and the ability to directly fabricate structures on unconventional surfaces like fiber end faces. Note that in contrast to wafer-based lithography, 3D nanoprinting is well suited for the fabrication of on-fiber nanostructures. The performance of the holograms is largely determined by the voxel dimension (300 nm transverse, 700 nm axial), which is a key factor in defining the spatial printing resolution. Therefore, the fs-DLW process ensures a high degree of accuracy in replicating the computationally designed phase profile, making it possible to translate theoretical designs into practical, high-performance holographic devices. To improve adhesion and prevent delamination, the fiber surface is silanized and plasma treated prior to printing, and a 10 *μ*m base layer of polymer is added to ensure a smooth, flat surface.

Optical characterization involves directing the excitation light (*λ*_0_ = 637 nm) into a selected core of a hologram-enhanced MCF and measuring the intensity distribution in the IP (details in Method section and supplementary note 4). Selective excitation of modes in specific cores is achieved using a custom microscope setup that projects images of the MCF input facet and the focused laser spot onto a camera, allowing selective excitation by adjusting the transverse position of the laser spot. The light intensity distribution at the focal plane is captured by another custom microscope setup.

**Fig. 2:**
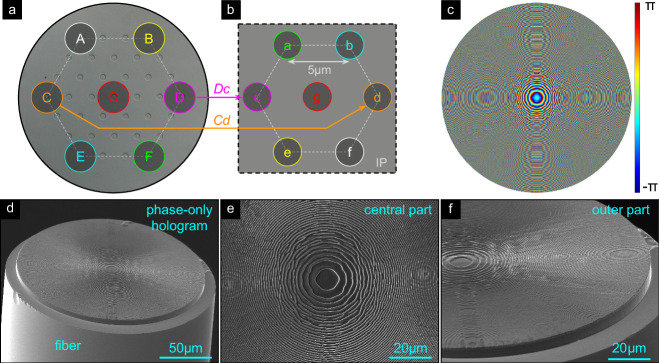
Design and realization of the hologram-functionalized SM-MCF for cross-axis focusing (conf_CF, e.g., *A* − > *f*). **a** SEM image of the SM-MCF surface (cores have been etched for better visibility). The circles (cyan) indicate the positions of the cores used in the GSA-design procedure (core labels: A-G). The dashed line indicates the hexagonal lattice. **b** Hexagonal arrangement of the considered foci (purple circles) in the IP (foci labels: **a**–**g**). To visualize the assignment of cores and focus positions (same color in (**a**) and (**b**)), two selected examples of core/focus position links are highlighted with arrows (orange: Cd, magenta: Dc). **c** Computational-designed phase hologram in HP (dimensions, *D*_h_ = 180 μm). The lower images show SEM images of the nanoprinted holograms on the SM-MCF ((**d**): Oblique view, (**e**): Top view of the central region of the hologram, (**f**): Tilted view of the edge region).

In the following, the feasibility of the concept of remote focus tuning via MCF-interfaced phase holograms is experimentally demonstrated by considering three different foci arrangements in the IP (conf_CF: cross-fiber-axis focusing, conf_SF: same-side focusing, conf_LF: line-array focusing). In Fig. 2 the center and outermost cores in the design are labeled with uppercase letters (A-G), while their corresponding foci are labeled with lowercase letters (a-g). The remaining cores are labeled according to their proximity to the nearest outer cores, starting from core G. For example, core GC1 refers to the core located between the center core (G) and the farthest core to the right (C), positioned in the immediate vicinity of core G. The exception is core GBD, which is located between cores G, B, and D.

#### Cross-axis focusing

The first design aims to create seven focal spots (focal length *f* = 50 μm, target *NA*_f_ ≈ 0.51) positioned on opposite sides of the central core axis (core “G”), resulting in cross-axis focusing (conf_CF, interfocus distance *Λ*_f_ = 5 μm; Fig. 2a, b). For example, the mode from core A is focused to position *f* in the IP (i.e., A  − > f). The retrieved phase distribution Φ(*x*, *y*) in the HP (Fig. 2c, obtained using the ASM-GSA procedure) shows an increasing spatial phase variation with distance from the center, resembling a kinoform profile. However, as will be shown later, a simple kinoform profile leads to significant aberrations, especially when the outer cores are excited, highlighting the relevance of the GSA design strategy. The phase distribution was converted into a corresponding height profile using Eq. (1) that was nanoprinted on an SM-MCF functionalized with a glass spacer (Fig. 2d, e). The resulting hologram (diameter *D*_h_ = 180 μm) closely matches the predicted profile, indicating a successful implementation of the phase distribution on the fiber.

**Fig. 3:**
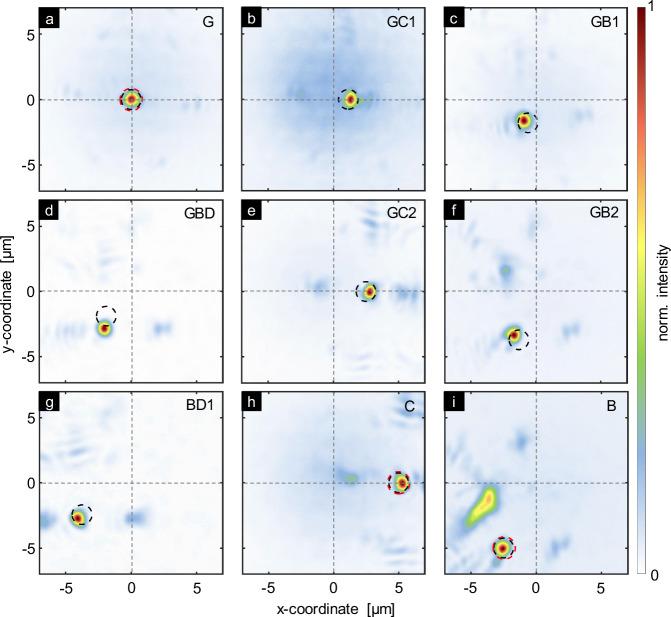
Characterization of cross-axis focusing. **a**–**i** show the measured spatial intensity distribution (conf_CF, linear scale) in the IP for modes excited in selected cores (core labels at top right of each plot). Intensities are normalized to their respective maximum values. Dotted lines indicate the coordinate system. Red dashed circles indicate the focus coordinates used in the hologram design, while black dashed circles indicate the simulated focus positions obtained when considering the hologram.

Experimentally, light was coupled into different cores of the hologram-enhanced SM-MCF and the resulting beam profiles were measured in the image plane (IP). Figure 3a–i shows a selection of measured intensity distributions, with the corresponding cores noted in top-right of each figure. Clearly visible in the images are locally concentrated light distributions, with the majority of the power localized at a specific location in the xy-plane, depending on the excited core. Note that outside the region of high-intensity concentration, the power is significantly lower and distributed over larger spatial areas, allowing a single focus to be clearly assigned to each excited core. The correlation between the excited core and the focus distribution is clearly visible in the arrangement of the measured focus positions. For example, excitation of the leftmost core (core C), the upper right core (core B), or the central core (core G) produces foci at *R*_C_ = (5 μm,0) (position d, Fig. 3h), *R*_B_ = (−2.5 μm, −5 μm) (position d, Fig. 3i), and *R*_G_ = (0,0) (position g, Fig. 3a), all of which correspond to the positions indicated in the pattern (red circles).

Interestingly, focal spots are also produced when intermediate cores, not included in the GSA design, are excited, showing highly local intensity distributions and much lower power outside these regions. For example, Fig. 3b, e, and h show a discrete focus shift in the positive x-direction, suggesting that the design procedure effectively allows to create foci for all cores within a single IP. Most cores focus at the intended and expected positions (red and black circles in Fig. 3). Deviations may be due to printing imperfections or misalignment of the hologram with the fiber axis.

**Fig. 4:**
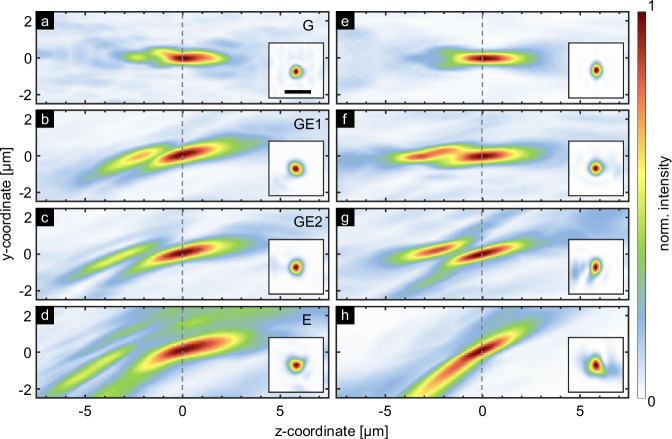
Comparison of experiment and simulation results for cross-axis focusing. The plot show the axial (yz-plane) and transverse (xy-plane, insets) intensity distributions (linear scale) in the focal region when the nuclei are excited along the connecting line between the central core (core G) and core E ((**a**–**d**): experiments, (**e**–**h**): corresponding ASM simulations). The scale in the inset in (**a**) refers to a distance of 1 μm. The dashed line corresponds to the target focal length of *f* = 50 μm, defined here at *z* = 0).

To evaluate the performance of the approach, Fig. 4 shows selected intensity distributions near the focal positions in two different orthogonal planes, comparing experimental results (left column) with simulations (right column, obtained by using ASM). All experimentally measured foci resemble the simulated light distribution, demonstrating the reliability of the focus generation by the presented concept. For quantitative analysis, key benchmark parameters have been extracted from this experimental data set (Table 1. The simulated NAs resulting from the final hologram (third row in Table 1) are slightly smaller than the design NA of 0.51 resulting from the superposition of individual holograms. Notably, the measured NAs (fourth row in Table 1) is very close to the simulated values, underscoring the high quality of the implemented structure.

**Table 1:** Summary of benchmark parameters extracted from the selected intensity distributions shown in Fig. 4 (conf_CF)

Core	G	GE1	GE2	E
theoretical numerical aperture	0.51	–	–	0.51
single hologram numerical aperture	0.51	–	–	0.41
GSA hologram numerical aperture	0.51	0.44	0.44	0.36
measured numerical aperture	0.50	0.39	0.40	0.35

The table shows the calculated and measured transverse numerical apertures for cores G, E, and intermediate cores. The first row shows the target *NA*_f_ = 0.51 used in the GSA to generate the final hologram. The second row shows the NA obtained from the individual hologram when excited by the mode in the corresponding core. The third and fourth rows are derived from simulations (Fig. 4e–h) and experiments (Fig. 4a–d).

**Fig. 5:**
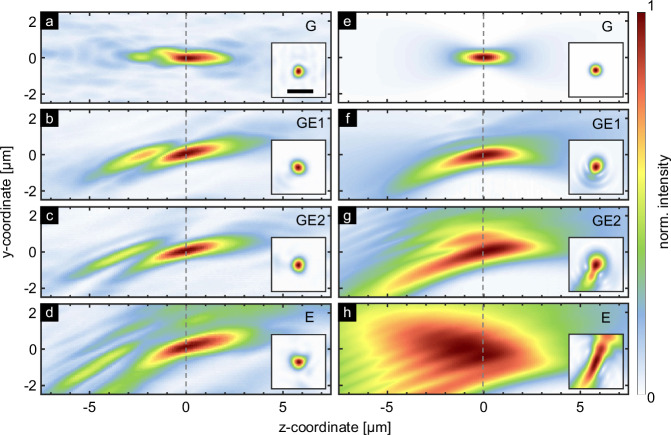
Comparison of experimental results to performance of kinoform profile. **a**–**d** Measured axial and transverse intensity distribution (linear scale) in the focal region for the cores addressed in Fig. 4 compared to (**e**–**h**) ASM-based beam propagation simulation assuming a kinoform phase profile obtained with the same design parameters (scale in inset in (**a**): 1 μm, dashed line: target focal length *f* = 50 μm).

To validate the use of the computer-designed phase hologram, the experimental results were compared with simulations of an equivalent lens that has a kinoform profile defined by the same design parameters (Fig. 5, *f* = 50 μm, *NA*_f_ = 0.51; see Fig. S4 in the supplementary note 5). Except for the central focus, the comparison shows that a kinoform profile alone is not sufficient to generate an accurate focus when off-axis cores are excited. This discrepancy confirms the need for computational phase design and a holographic approach.

#### Same-side focusing

**Fig. 6:**
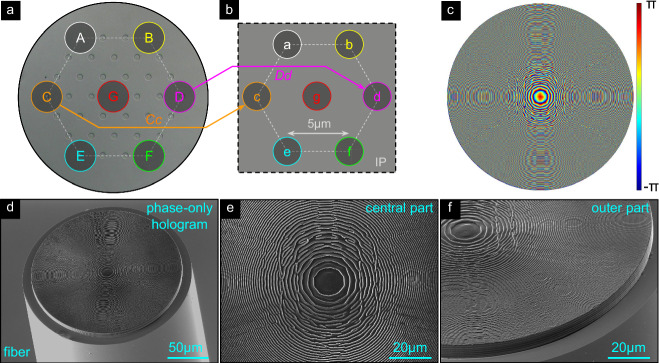
Design and realization of the hologram-functionalized SM-MCF for same-side focusing (conf_SF, e.g., A  − > a). **a** SEM image of the SM-MCF surface, with cyan circles indicating the positions of the cores (core labels A-G). Dashed line: hexagonal lattice. **b** Arrangement of the foci (purple circles) in the IP (foci labels: **a**–**g**). To illustrate the assignments of core and focus positions (same colors in (**a**) and (**b**)), two examples are marked with arrows (orange for Cc, magenta for Dd). **c** Retrieved phase hologram (diameter *D*_h_ = 180 μm). Bottom row: SEM images of the nanoprinted holograms on the fiber: **d** oblique view, **e** top view of the central region of the hologram, and **f** oblique view of the edge region.

**Fig. 7:**
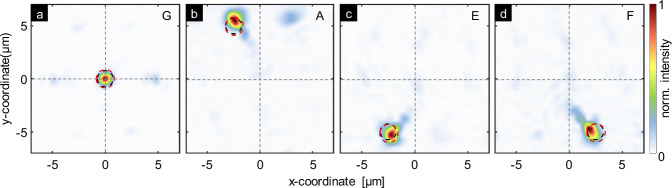
Experimental characterization of same-side focussing. **a**–**d** show the measured spatial intensity distribution (conf_SF, linear scale) in the IP for modes excited in selected cores (core labels at top right of each plot). Intensities are normalized to their respective maximum values. Dotted lines indicate the coordinate system. Red dashed circles show the focus coordinates used in the hologram design, while black dashed circles indicate the simulated focus positions obtained when considering the hologram.

The second configuration (conf_SF) involves an arrangement of foci that resembles the core distribution, resulting in same-side focusing (Fig. 6a–b, target focal length *f* = 50 μm and *NA*_f_ = 0.51). For example, light from core A is focused at point a in the IP at coordinates *R*_A_ = (−2.5 μm, 5 μm) (A  − > a). Similar to the previous procedure, the phase distribution was retrieved using the ASM-GSA approach (Fig. 6c), and the design was experimentally implemented using 3D nanoprinting on the end face of the spacer (Fig. 6d–f). To evaluate the performance, laser light was selectively coupled to the six most outer cores and the central core (design cores), and the resulting intensity distributions in the IP were retrieved (Fig. 7a–d). Each image shows a strong local concentration of intensity resembling a focal spot, while outside these regions, light is barely visible and unevenly distributed. Note that in this configuration, the 37-core fiber effectively operates as a 7-core MCF with an inter-core pitch of 45 *μ*m. We chose this approach, as producing a new 7-core fiber with identical functionality to the 37-core fiber solely operating with the design codes would require additional time, manufacturing effort, and resources. The design (red dashed circles), simulations (blue dashed circles), and experimental results show strong agreement, verifying the use of the design procedure and the quality of the implemented structures. Note that same-side focusing cannot be achieved with a conventional hyperbolic phase profile lens, demonstrating the relevance of using a holographic approach to achieve such functionality.

#### Line-array focusing

**Fig. 8:**
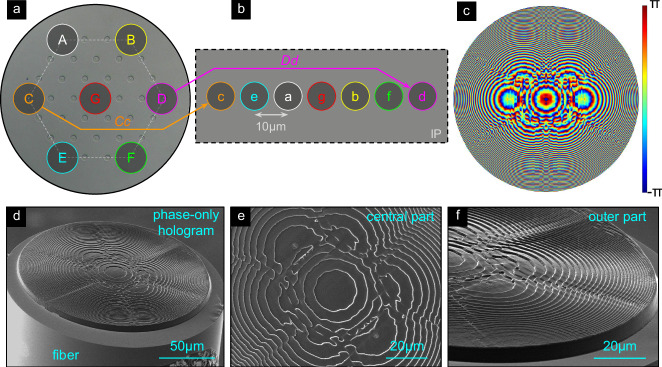
Design and realization of the line-type focus arrangement (conf_LF). **a** Surface of the SM-MCF with circles (cyan) indicating the positions of the cores (A-G: core labels). **b** Line arrangement of the foci (purple circles) in the IP (a-g: focus labels). Similar to the previous figures, linked cores and focus positions share the same colors (two example assignments are highlighted by the arrows). **c** Calculated phase hologram in HP (dimensions, *D*_h_ = 180 μm). Bottom row: SEM images of the nanoprinted holograms on SM-MCF (**d**: Oblique view, **e**: Top view of the central region of the hologram, **f**: Tilted view of the edge region).

The third configuration (conf_LF) arranges the foci along a straight line. Here, cores A-G are considered (Fig. 8a), with seven foci (Fig. 8b, target focal length *f* = 197 μm and *NA*_f_ = 0.15) spaced 10 *μ*m apart. The resulting hologram (Fig. 8c), designed by the ASM-GSA approach, is more complex compared to the previous designs (Figs. 2c and Fig. 6c) and shows indications of the GBs in the phase distribution. Visual inspection of the nanoprinted structures on the SM-MCFs (Fig. 8d) shows strong agreement with the computed hologram, especially in the finely structured central region (Fig. 8e) and the edge region (Fig. 8f), where rapid phase oscillations occur.

**Fig. 9:**
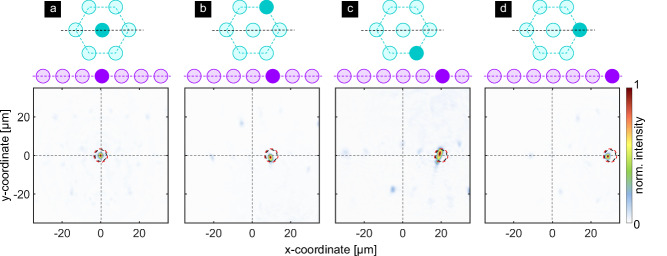
Experimental characterization of line-array focussing. **a**–**d** show examples of measured intensity distributions of the line-array focusing configuration (conf_LF). Each plot corresponds to a combination of addressed core and foci. To emphasize this correlation, the top, and middle rows visualize the addressed core in the SM-MCF (Fig. 8a) and the corresponding foci in the array (Fig. 8b). The black dashed lines in the top row indicate the horizontal symmetry line of the core distribution. The bottom row shows the measured intensity distribution (normalized to the corresponding maximum). The red dashed circles refer to the focus coordinates used in the hologram design. The black dashed circles show the simulated focus positions obtained when considering the hologram.

Experimental characterization relying on launching light into the six most outer cores and the central core (design cores) using the same procedure as for previous configurations shows that single spots of highly localized intensity can be experimentally generated along a horizontal line (c.f. plots in the lower row of Fig. 9). The corresponding positions match those defined by simulations and design (red and blue dashed circles), with a maximum shift of the focus from the center position of 29 μm. The intensity outside these areas of high field concentration is relatively low and unevenly distributed, indicating that a clear correlation between the addressed core and the location of the corresponding focus can be achieved. Note that due to the complex correlation between the foci and their associated cores, slight deviations from the linear arrangement and a reduced symmetry of the foci intensity distributions are observed for the foci generated by off-axis cores (i.e., cores not aligned with the horizontal dashed lines in Fig. 9). Similar to the previous configuration (conf_SF), the 37-core fiber functions as a 7-core MCF with an intercore pitch of 45 μm.

## Discussion

The hologram-enhanced SM-MCF presented here opens up several potential avenues for future research. One direction is to explore the generation of complex images rather than focus generation or additional phase control within the generated intensity patterns, which is relevant for exciting resonant optical states (e.g., fiber modes or nanophotonic resonances). Another direction is to increase the number of cores within the fiber, with the most prominent example being the Fujikura Imaging Fiber^41^. Further developments in optimization algorithms and machine learning can also significantly improve the generation of phase holograms, leading to improved performance. The concept could also benefit from advances in nanoprinting technology, such as STED-based nanoprinting^42^ or grayscale lithography^43^. Future research could explore the use of complex nanostructures such as metasurfaces that involve polarization manipulation. Initial promising experiments in this area have been conducted^21^, demonstrating the potential for complex on-fiber beam manipulation. In addition to the single-core excitation presented in this work, spatial light modulators (SLMs) or digital micromirror devices (DMDs) could potentially be used to excite mode in multiple cores simultaneously, providing an additional degree of freedom for research exploration. This offers exciting possibilities for creating complex beams with tailored properties, as the SLM allows precise phase or amplitude control of the output light of each core. For example, as demonstrated in ref.^33^, the coherent combination of multiple tailored GBs from the cores of an MCF amplifier facilitates the creation of complex beam types. Here our concept could potentially integrate beam-combining optics directly onto the fiber end face, paving the way for an all-fiber device capable of producing higher-order linearly polarized modes, cylindrical vector beams, and optical vortex beams with helical phase fronts. The combination of the holographic lenses concept and MCFs may also be used to reveal nonlinear pulse dynamics in MCF systems (e.g., ref.^37^). Moreover, integrating SLMs into our concept enhances the flexibility and functionality of our metafiber system by enabling precise core excitation without mechanical adjustments. Note that current investigations are aimed at developing methods for creating reconfigurable focal positions, including responsive holograms and other adaptive approaches, to increase flexibility in applications requiring dynamic focus control.

In addition to the phase-only hologram, a key advantage of the concept presented here is SM-MCF that is single-mode at visible wavelength and thus is insensitive to bending. This is in stark contrast to systems using multimode fibers, where bending changes the transmission matrix, affecting the output beam profile and consequently focusing characteristics. In contrast, the SM-MCF concept is substantially less susceptible to bending, making it suitable for a wide range of applications. The use of 37 cores greatly increases the flexibility of beam control, allowing a large number of foci to be generated that would be limited in case fewer cores are considered. In addition, the hexagonal core arrangement maximizes core density while maintaining negligible modal crosstalk and ensures uniform core dimensions during fiber drawing, unlike linear core arrangements that can lead to uneven force distribution and core variability during fiber implementation.

The use of fs-DLW for on-fiber 3D nanoprinting is critical for this work, enabling the precise fabrication of on-fiber holograms with a level of detail and accuracy that directly translates into superior optical performance. This technique not only supports the implementation of complex holographic designs but also allows for future scalability to more intricate or application-specific hologram structures. The ability to fabricate such detailed nanostructures on small and unconventional surfaces such as a fiber end face showcases the potential of fs-DLW to advance integrated photonic devices and unlock new functionalities in optical systems.

The concept of remote focus manipulation using a computationally designed hologram interfaced with SM-MCF shows great promise in several applications. In high-speed laser micromachining, the ability to quickly adjust the focus increases both the precision and the throughput of manufacturing processes^44^. In optical trapping and manipulation, it enables precise, rapid particle control without mechanical components, making it ideal for biological and microscopic applications^45^. The system could also significantly improve optical signal processing in telecommunications by optimizing mode control in fiber networks^46^ and improving in-coupling in integrated photonics^47^. Another potential application is the coherent combination of multi-core fiber laser outputs into a single beam^36^, achieved by enabling precise phase and focus control at the output of each core via computationally designed holograms. Note that additional phase control across beams emitted from multiple cores could be achieved by careful design of the nanoprinted holograms (c.f.^19^). Moreover, precise and rapid focus modulation could significantly enhance laser surgery, providing greater accuracy and improved safety^48^.

In this study, we introduce and experimentally validate an unexplored concept for remote focus manipulation using computationally designed 3D nanoprinted holograms on the end face of single-mode multi-core fibers. This approach allows light coupled into any of the 37 cores to be precisely focused at a predefined location in the image plane, enabling advanced remote light control within a monolithic meta-fiber device. Our method, which uses computationally designed holograms that closely match predicted profiles, allows for the implementation of multiple configurations with functionalities beyond that of conventional lenses, highlighting the effectiveness of the holographic approach. This work marks the first demonstration of the use of multi-core fibers for nanostructure-empowered remote focus control via 3D nanoprinting, with a fiber design featuring a record number of cores designed for crosstalk-free operation at visible wavelengths. Key achievements of the study include extensive optical characterization confirming excellent agreement between design, simulation, and experimental results, computational design of holograms using a modified Gerchberg-Saxton algorithm, and precise on-fiber implementation via advanced 3D nanoprinting technology. Overall, this innovative monolithic all-fiber device offers a new level of remote focus control and has the potential to outperform conventional devices in a wide range of applications, including optical manipulation for biology and microscopy, high-speed laser micromachining, telecommunications, integrated photonics, and laser surgery. The promising results of this study pave the way for the development of advanced optical systems.

## Methods

### Implementation of the holographic metalens on single-mode multicore fiber

#### Properties of the nano printing process

The 3D laser nanoprinting of polymer-based metalens relies on two-photon lithography-based femtosecond direct laser writing (fs-DLW) using a commercial photolithography system (Photonic Professional GT, Nanoscribe GmbH). The fabrication is a mask-less technique that relies on two-photon absorption-based polymerization via the formation of subwavelength ellipsoidal building blocks called voxel cells (dimensions 300 nm x 700 nm). This approach offers advantages such as speed, reliability, and cost efficiency, and enables the direct realization of nanostructures on optical fibers without additional post-processing or clean room requirements.

#### Fiber surface activation

Before nanoprinting, the fiber facet was chemically activated in a two-step process involving (i) oxygen plasma activation applied for 1 min (150 W), (ii) followed by silanization based on overnight immersion in 1% 3-(trimethoxysilyl)propyl methacrylate (Sigma-Aldrich 440159) dissolved in ethanol and nitrogen drying. Prior to the first step, the fiber tip was cleaned by successive sonication in acetone, isopropyl alcohol, and distilled water (15 min each), followed by nitrogen drying.

#### Nanoprinting

To align the MCF within the nano printer, light was coupled into the cores from the unpatterned end of the fiber, allowing the initial position (*x*, *y*, and *z* coordinates) of the fiber and cores to be identified using a low magnification (Plan-Apochromat 20x/0.85, Zeiss) air lens. The negative photosensitive IP-Dip (Nanoscribe GmbH) was then dropped onto the fiber and the translation stage was moved to predetermined 3D coordinates under a high numerical aperture objective (Plan-Apochromat 63x/1.40 oil DIC, Zeiss). After finding the fiber end face under the high numerical aperture objective, 3D laser nanoprinting was started. Note that a 10 μm thick base layer was first printed to improve adhesion between lens and fiber and to ensure a flat and smooth surface. Then the actual printing of the metalens over the polymer base was started (50 nm hatching, 100 nm slicing, about 3 h printing time). For optimal printing, a laser power of 12.5 mW and a scan speed of 8000 μm/s were used. After laser exposure, the structure was developed in a propylene glycol monomethyl ether acetate (PGMEA, Sigma-Aldrich 484431) bath for 25 min, followed by a 3 min Methoxy-nonafluorobutane (Novec 7100 Engineered Fluid, 3M) rinse. The final sample was rinsed with isopropyl alcohol prior to measurement.

### Optical characterization

Optical characterization involves directing light from a continuous wave laser diode (Thorlabs LP637-SF70, *λ*_0_ = 637 nm) into a selected core of the hologram-enhanced MCF and measuring the intensity distribution in the IP. Selective mode excitation within a specific core was facilitated by a custom microscope setup consisting of an objective (Olympus UPlanFL N 20x) and a lens (Thorlabs AC254-300-A-ML). This setup projects an image of the MCF input facet together with the launching laser spot onto a CMOS camera (Thorlabs DCC1545M), allowing precise selection of the core to be excited by moving the laser spot transversely across the MCF surface. The intensity distribution in the focal plane was captured by a custom-built microscope (Olympus MPlanFL N 50x + Thorlabs AC254-300-A-ML) connected to a CMOS camera (Thorlabs DCC1545M).

## Supplementary information

Supplementary Information

Transparent Peer Review file

## Data Availability

The data that support the findings of this study are available from the corresponding author upon request.

## Code availability

We employed the POV-Ray (Persistence of Vision Raytracer, version 3.7) framework^49^ to produce Fig. 1a. Custom codes used to generate results reported in the manuscript can be made available from the corresponding upon request.
