# Supplementary material for: Advanced remote focus control in multicore meta-fibers through 3D nanoprinted phase-only holograms
Source: Nat Commun. 2025 Jan 8;16:507. doi: 10.1038/s41467-024-55805-7 (PMC11711281; doi:10.1038/s41467-024-55805-7)
Supplement: Supplementary file 1 — Supplementary Information [file 41467_2024_55805_MOESM1_ESM.pdf]

Supplementary information for

Advanced remote focus control in multicore meta-fibers through 3D nanoprinted phase-only holograms

MOHAMMADHOSSEIN KHOSRAVI[1,2], TORSTEN WIEDUWILT[1], MATTHIAS ZEISBERGER[1], ADRIAN LORENZ[1], MARKUS A. SCHMIDT[1,2,3*]

[1]Leibniz Institute of Photonic Technology, 07745 Jena, Germany.
[2]Abbe Center of Photonics and Faculty of Physics, FSU Jena, 07745, Jena, Germany.
[3]Otto Schott Institute of Material Research, FSU Jena, 07745, Jena, Germany.

**Corresponding author(s). E-mail(s): markus.schmidt@ipht-jena.de*

This document presents the supplementary details in the main manuscript.

Supplementary Note1. PROPERTIES OF THE SM-MCF

The mode field diameter (MFD) and numerical aperture (NA_{MCF}) were determined for selected cores by analyzing the diffraction of the beam in air with images taken at various distances from the fiber surface. Figure S1 shows two examples of the measured beam profiles for the central core (top) and a selected outer core (bottom), both fitted by a Gaussian beam model. The fitting parameters yielded very similar values for both cores (5.2 µm and 5.3 µm), resulting in nearly identical numerical apertures ($NA_{\mathrm{MCF}} = 0.08$). This consistency serves as an indicator of the quality of the SM-MCF and allows the use of the same Gaussian beam model in the hologram design procedure.

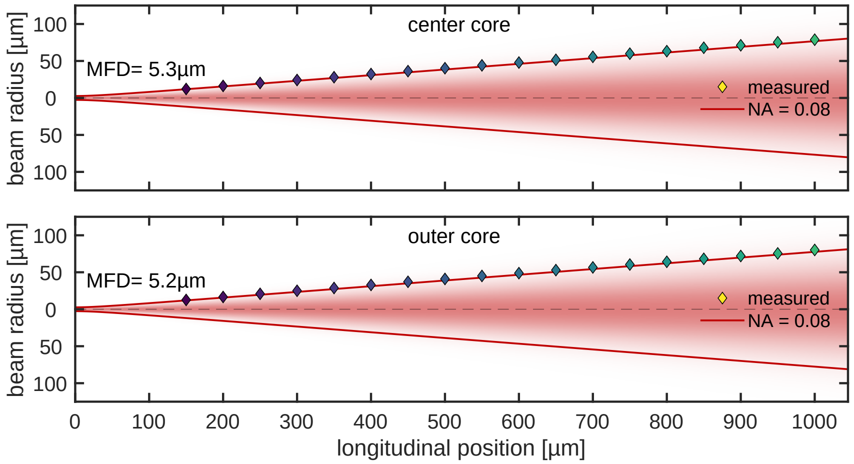

Fig. S1. Measured beam profile at different distances to the fiber surface along the axial direction (top: central core, bottom: selected outer core). The diamonds represent the measurement points, while the red lines correspond to fits modeled as GBs. The gradually changing red color visually indicates the intensity distribution of the beam along its path.

Supplementary Note2. PHASE RETRIEVAL USING THE GERCHBERG-SAXTON ALGORITHM (GSA)

In this study, the phase distribution in the HP was determined using the Gerchberg-Saxton Algorithm (GSA), which iteratively refines phase estimates by alternating between the hologram plane (HP) and the image plane (IP). The GSA works by performing forward and backward light propagation between these planes, using known intensity distributions in each plane to progressively converge on an accurate phase solution. Initially, the intensity distribution in the HP commonly uses a random phase profile, while we found that a kinoform $\left(\Phi_{\text{init}} = \text{mod}\left(2\pi, \Phi_{\text{hyp}}\right)\right.$ with the hyperbolic phase profile $\Phi_{\text{hyp}} = -nk_0\left(\sqrt{r^2+f^2}-f\right)$, r: radial coordinate, f: focal length) phase profile results in a more symmetric output and faster convergence. The GSA workflow is as follows (Fig. S2):

1. **Initialization:** The process begins by assigning a kino-form phase profile to the intensity distribution at the fiber end face.
2. **Forward Propagation:** Using the Angular Spectral Method (ASM), the complex field in the HP is propagated forward and the corresponding complex field in the HP is calculated.
3. **Intensity Substitution:** The calculated intensity in the HP is replaced by the desired intensity pattern, which in the present case are the focal points defined by Airy functions.
4. **Backward Propagation:** The updated complex field undergoes backward propagation, refining the phase distribution at the HP.
5. **Iteration:** The intensity in the HP is replaced by the known intensity defined by the corresponding GBs.

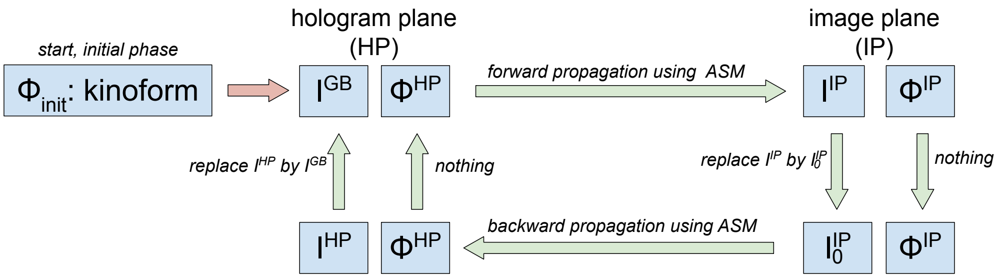

Fig. S2. Working principle of the Gerchberg-Saxton algorithm used to obtain the phase distribution in the HP for a single combination of GB and focus location in the IP.

This cycle is repeated until the output intensity closely matches the desired pattern, ensuring convergence of the algorithm, with the GSA individually applied to each of the seven GBs. As outlined in the main text, the resulting fields were then combined to compute a unified phase pattern at the fiber facet.

Supplementary Note3. ANGULAR SPECTRUM METHOD

In this study, the angular spectrum method (ASM) was used within the GSA to calculate both forward propagation (from HP to IP) and backward propagation (from IP to HP). This method is particularly suited to the problem addressed in this manuscript, as it allows for the calculation of beam propagation over arbitrary and in particular short distances, including very short ones. ASM achieves this flexibility by decomposing the field into independently propagating plane waves, each characterized by a specific wave vector [1]. This approach is crucial for accurately determining the phase distribution required to achieve precise focal points at the desired distances. Conventional approaches such as Fraunhofer or Fresnel diffraction are inadequate for modeling focal distances on the order of a few tens of microns, which are the focus of this study.
The ASM is based on the convolution theorem and can be used to calculate the scalar complex

field $U(X,Y)$ at a distance z in the IP from a known field $u(x,y)$ in the HP. The relationship between the input and output planes is governed by the following equation:

$$U(X,Y) = \mathcal{F}^{-1}\left\{\mathcal{F}[u(x,y)] \cdot H(f_x, f_y)\right\} \tag{S1}$$

Here, F and $\mathcal{F}^{-1}$ represent the Fourier and inverse Fourier transforms, respectively. The coordinates (X,Y) and (x,y) refer to positions in the HP at $z = 0$ and IP at distance z from the HP, while (f_x, f_y) denote the corresponding spatial frequency coordinates. The propagation transfer function, $H(f_x, f_y)$ for a distance z is given by:

$$H(f_x, f_y) = \exp\left(-ikz\sqrt{1 - (\lambda f_x)^2 - (\lambda f_y)^2}\right) \tag{S2}$$

where $k = 2\pi/\lambda_0$ is the wavenumber, and λ_0 is the wavelength. The forward propagation of the complex field can thus be computed by applying the above equation, leading to the field distribution at the desired IP.
For backward propagation, the reverse process is applied, using the following equation:

$$u(x,y) = \mathcal{F}^{-1}\left\{\mathcal{F}[U(X,Y)] \cdot H^*(f_x, f_y)\right\} \tag{S3}$$

Here, $H^*(f_x, f_y)$ represents the complex conjugate of the propagation transfer function, allowing us to retrieve the phase distribution at the HP after back-propagating the field from the IP.

Supplementary Note4. EXPERIMENTAL SETUP FOR BEAM CHARACTERIZATION

The setup used for the experimental characterization is composed of two main parts (Fig. S3) which are explained individually in the following.

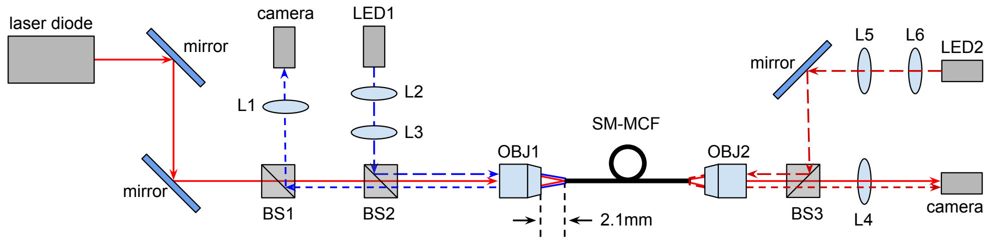

Fig. S3. Sketch of the experimental setup used to characterize the intensity distribution in the IP crested by the hologram-enhanced MCF.

Input side: The first part, situated on the left side of the MCF, serves to couple light from the laser diode into one of the cores while simultaneously imaging the fiber's input facet. The 20x magnification of the objective lens (OBJ1) allows us to capture the entire surface of the MCF, while its 0.5 numerical aperture (NA) enables single-core coupling, with minimal light being coupled into the other cores.

Output side: The second part, located on the right side of the MCF, is designed to image the output face of the MCF and evaluate the foci created by the hologram. The high NA of the second objective (OBJ2) provides high resolution for imaging the foci and the image plane. Since a monochrome camera is used, the similar colors of the laser beam and illumination do not interfere with the evaluation.
The following table includes the specific items used to create the setup.

Item	Specification
Laser Diode	Thorlabs LP637-SF70 - 637 nm
Beam Splitter 1 (BS1)	Thorlabs CCM1-BS013
Beam Splitter 2 (BS2)	Thorlabs CCM1-BS013
Beam Splitter 3 (BS3)	Thorlabs CCM1-BS013
Objective Input (OBJ1)	Olympus UPlanFL N 20x / 0.5NA
Objective Output (OBJ2)	Olympus MPlanFL N 50x / 0.8NA
Camera	Thorlabs DCC1545M (same at input and output)
LED1	Thorlabs M455F3 - 455 nm
LED2	Thorlabs M617F2 - 617 nm
L1	Thorlabs AC254-300-A-ML
L2	Thorlabs AC254-040-A-ML
L3	Thorlabs AC254-150-A-ML
L4	Thorlabs AC254-300-A-ML
L5	Thorlabs AC254-300-A-ML
L6	Thorlabs AC254-030-AB-ML

Wavelength to be coupled: 637nm
Wavelength illumination input: 455nm
Wavelength illumination output: 617nm

Supplementary Note5. EQUIVALENT KINOFORM PROFILE

The results of the cross-axis focusing configuration (conf_CF) were compared with simulations that included a kinoform profile obtained using the defined target numerical aperture ($NA = 0.51$) and focal length ($f = 50\mu m$). This profile (shown in Fig. SS4) is calculated using the hyperbolic phase profile in connection with the modulus of 2π [2].

$$\Phi_{\text{hyp}}(x,y) = -2\pi n/\lambda_0 \left(\sqrt{r^2+f^2} - f\right) \tag{S4}$$

$$\Phi_{\text{kino}}(x,y) = \text{mod}\left(2\pi, \Phi_{\text{hyp}}\,(x,y)\right) \tag{S5}$$

with the refractive index of the environment n and the radial coordinate r.

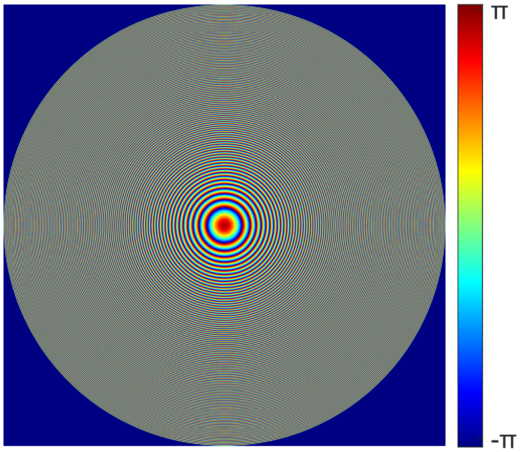

Fig. S4. Phase distribution of the kinoform that has identical design parameters than used for the calculation of the phase-only hologram in the context of the cross-axis configuration (conf_CF, Fig. 5).

LIST OF KEY SYMBOLS

Symbol	Unit	Description
N_{c}	1	No. cores inside the MCF
N_{h}	1	No. cores used for the design of the hologram
f	m	Focal length
λ_0	m	Operation wavelength (in vacuum)
Φ	-	Phase distribution of the hologram (designed and corrected)
Λ_{des}	m	Design inter-core spacing (most outer cores and central core)
Λ_{f}	m	Designed distance between foci
R	m	Focus coordinates
D_{h}	m	Diameter of hologram on fiber surface
L_{s}	m	Length of the glass spacer
NA_{MCF}	1	Numerical aperture of the cores in the multicore fiber
NA_{f}	1	Numerical aperture of the individual focus

Table S1. Table of symbols, units, and descriptions

REFERENCES

1. B. E. Saleh and M. C. Teich, *Fundamentals of photonics* (John Wiley & Sons, 2019).
2. M. Plidschun, H. Ren, J. Kim, *et al.*, "Ultrahigh numerical aperture meta-fibre for flexible optical trapping," Light. Sci. & Appl. **10**, 57 (2021).
