# Supplementary material for: Advanced remote focus control in multicore meta-fibers through 3D nanoprinted phase-only holograms
Source: Nat Commun. 2025 Jan 8;16:507. doi: 10.1038/s41467-024-55805-7 (PMC11711281; doi:10.1038/s41467-024-55805-7)
Supplement: Supplementary file 2 — Transparent Peer Review file [file 41467_2024_55805_MOESM2_ESM.pdf]

nature portfolio

Peer Review File

Advanced remote focus control in multicore meta-fibers through 3D nanoprinted phase-only holograms

Corresponding Author: Professor Markus Schmidt

This file contains all reviewer reports in order by version, followed by all author rebuttals in order by version.

Version 0:

Reviewer comments:

Reviewer #1

(Remarks to the Author)
The paper presents a novel concept of nanoprinting 3D holograms on the end face of singlemode multicore fibers for the sake of remote focus manipulation. In particular, it allows for the light coupled into any of the 37 cores to be precisely focused at a predefined location in the image plane. The computationally designed holograms using a modified Gerchberg-Saxton algorithm closely match predicted profiles, three types of holograms nanoprinted on the fiber end (coreless fiber spacer attached to the multicore fiber) show good agreement with experimentally measured focus positions in three configurations (cross-fiber-axis focusing, same-side focusing, line-array focusing).

This monolithic all-fiber approach really has the potential to outperform conventional devices in a wide range of applications, including optical manipulation for biology and microscopy, high-speed laser micromachining, telecommunications, integrated photonics, and laser surgery.

The manuscript definitely deserves publication in Nature Comm. after clarification of the following issues:
1) Is it possible to apply this approach for coherent combining of multicore fiber laser output (into a single beam)?
2) Is it possible to combine this approach with selective coupling of the input beam to a selected core with the use of spatial light modulator(SLM) similar to that in papers [a, b]? Please discuss this in more detail, as well as a potential of using SLM for the focusing output beams from a multicore fiber.
3) Please discuss in more detail the limitations of the technology, for example, single hologram provides fixed positions of the focal spots from individual core beams. May the proposed approach to be modified somehow to become reconfigurable that important for many applications.
[a] D. Lin et al. Nat. Comm. 11(1), 1–9 (2020).
[b] D.S. Kharenko et al. Proc. SPIE, vol.12775: Quantum and Nonlinear Optics X, 127750W (2023) https://doi.org/10.1117/12.2688542

Reviewer #2

(Remarks to the Author)
In this paper, the authors present an approach for remote focus manipulation using 3D nanoprinted holograms integrated on the end face of multi-core single-mode fibers. This method enables precise focus control within a monolithic metafiber device by allowing light coupled into any of the 37 cores to be precisely focused at predefined locations. The authors mention this research marks the first successful use of multi-core fibers for remote focus control via 3D nanoprinting, achieving crosstalk-free operation at visible wavelengths. Key findings include strong agreement between design, simulation, and experimental results, highlighting the potential of this technology to improve applications in fields such as biological optics, laser micro-machining, telecommunications, and laser surgery. This article is clear, concise, and suitable for the journal's scope. Several suggestions are supplied:
1. Suggest the authors enhance the introduction part, such as the background also the motivation for this work.
2. The authors use 37 GeO_2-doped core, any specific reason?
3. The authors perform the 3D nanoprinting of on-fiber holograms using femtosecond direct laser writing, suggesting the authors enhance the achievement of this work.

4. Suggest the authors supply more detail in sentences about measured spatial intensity distribution (conf CF, linear scale) in the IP for modes excited in selected cores (core labels at the top right of each plot).
5. Suggest the authors supply more detail in sentences about the results of examples measured intensity distributions of the line-array focusing configuration.
To conclude, this work has merit and also has the potential to improve some applications.

Reviewer #3

(Remarks to the Author)
The manuscript “Advanced remote focus control in multicore meta-fibers through 3D nanoprinted phase-only holograms” by Khosravi et al. reports on the fabrication of a phase-only hologram on the end facet of a multicore optical fiber that allows for remote focusing depending on the excited core.
The paper's concept is novel and clever; it will surely have a large impact. As such, it meets all the high-standard criteria for publication in Nature Communication.
The results are beautiful and interesting. By reading the manuscript, one may really feel the care taken to achieve the efficiency of the nanostructure fabrication process. Still, before recommending the manuscript publication, I have a few comments that I would like to Authors to consider.
1. The first question that came to my mind when reading the manuscript was “Why use 37 cores if, eventually, one only gets 7 focusing points?”. I feel that this aspect is crucial and still not discussed in the manuscript. Specifically, is a mismatch between the number of cores and the number of focusing points (i.e., holograms) needed, and why?
2. Overall, I believe that the quality of the presentation can be improved. The labeling of the foci and the nanostructures’ positions may be hard to follow if not strongly supported with clear figures. I noticed, for instance, that Fig. 2(a-b) and Fig. 6(a-b) are identical. This makes it hard to understand which cases they refer to. In my opinion, an extra panel showing connecting arrows between the nanostructures’ positions with the foci positions will be beneficial to improve the presentation of the results.
Minor points
3. In line 31, a closing parenthesis “)” is missing after the word “ablation”.
4. In line 34, the Authors specifically refer to step-index fibers. I believe that all types of optical fibers are efficient solutions for light transportation. No need to specify “step-index”. In addition, in line 35, it is written that step-index fibers have doped core and undoped cladding. This is not always true. Often, indeed, step-index fibers are made of undoped silica core and Fluorine-doped silica cladding.
5. In the introduction, there is quite an abrupt jump from line 38 to line 39. Perhaps, the Authors may consider sharpening it a little by saying something like “When it comes to the control of light focusing, a key area of photonic research is…”.
6. The sentence “As a result, by exciting the mode of a specific core, the focus position can be dynamically controlled within the IP.” in line 94 is not clear to me. The expression “the mode of a specific core” is confusing since each core supports only one mode. Moreover, I do not understand how the dynamic control of the focus position results from the incorporation of the coreless fiber section.
7. In line 115, it is specified, for the first time, that the nanostructure is made of a polymer material. I believe that this should be stated above.
8. The acronym “GB” is defined twice.

Version 1:

Reviewer comments:

Reviewer #1

(Remarks to the Author)
I feel that all the questions raised by the reviewers are properly discussed by the authors, so I recommend the manuscript for publication as is.

Reviewer #2

(Remarks to the Author)
The authors improved the draft with the reviewers' comments, so I can now recommend that it be published in Nature Communication.

Reviewer #3

(Remarks to the Author)
All my concerns have been properly addressed.
The new version of the manuscript is suitable for publication in Nature Communications.

Open Access This Peer Review File is licensed under a Creative Commons Attribution 4.0 International License, which permits use, sharing, adaptation, distribution and reproduction in any medium or format, as long as you give appropriate credit to the original author(s) and the source, provide a link to the Creative Commons license, and indicate if changes were made.
In cases where reviewers are anonymous, credit should be given to 'Anonymous Referee' and the source.
The images or other third party material in this Peer Review File are included in the article's Creative Commons license, unless indicated otherwise in a credit line to the material. If material is not included in the article's Creative Commons license and your intended use is not permitted by statutory regulation or exceeds the permitted use, you will need to obtain permission directly from the copyright holder.
To view a copy of this license, visit https://creativecommons.org/licenses/by/4.0/

Response to comments of Reviewer #1

The paper presents a novel concept of nanoprinting 3D holograms on the end face of singlemode multicore fibers for the sake of remote focus manipulation. In particular, it allows for the light coupled into any of the 37 cores to be precisely focused at a predefined location in the image plane. The computationally designed holograms using a modified Gerchberg-Saxton algorithm closely match predicted profiles, three types of holograms nanoprinted on the fiber end (coreless fiber spacer attached to the multicore fiber) show good agreement with experimentally measured focus positions in three configurations (cross-fiber-axis focusing, same-side focusing, line-array focusing).

This monolithic all-fiber approach really has the potential to outperform conventional devices in a wide range of applications, including optical manipulation for biology and microscopy, high-speed laser micromachining, telecommunications, integrated photonics, and laser surgery.

We appreciate the Reviewer's detailed review, the positive assessment and the recommendation to publish this work in Nature Communications. Detailed responses to each of the comments are provided in the following sections of this letter.

The manuscript definitely deserves publication in Nature Comm. after clarification of the following issues:

R1.1: Is it possible to apply this approach for coherent combining of multicore fiber laser output (into a single beam)?

We thank the Reviewer for raising this important point regarding the application of the concept in the context of fiber lasers. We believe that our approach could, in principle, be used for coherent combining by providing precise phase and focus control at the output of each core through computationally designed holograms. Note that by modifying our hologram design, additional phase control between the beams emitted by the multiple cores can be realized to possibly focus or superimpose the outputs into a single coherent beam (c.f. [link]). This would require further development to ensure stable control, possibly through feedback control mechanisms or the employment of a spatial light modulator, as discussed in the next comment of the Reviewer.

To account for the Reviewer's comment, this point has been addressed by the following modification of the main text of the manuscript: … The system could also significantly improve optical signal processing in telecommunications by optimizing mode control in fiber networks [REF] and improving in-coupling in integrated photonics [REF]. **Another potential application is the coherent combination of multi-core fiber laser outputs into a single beam [link], achieved by enabling precise phase and focus control at the output of each core via computationally designed holograms. Note that additional phase control across beams emitted from multiple cores could be achieved by careful design of the nanoprinted holograms (c.f. [link]).** Moreover, precise and rapid focus modulation could significantly enhance laser surgery, providing greater accuracy and improved safety [REF]. …

R1.2: Is it possible to combine this approach with selective coupling of the input beam to a selected core with the use of spatial light modulator (SLM) similar to that in papers [a, b]? Please discuss this in more detail, as well as a potential of using SLM for the focusing output beams from a multicore fiber.

We thank the Reviewer for this insightful point regarding the employment of spatial light modulators (SLMs) for mode excitation within the multicore fiber and the two studies mentioned. We see two main advantages:

Increased flexibility in light coupling: The integration of SLMs into our concept offers a promising way to increase the flexibility and functionality of our metafiber system. By selectively coupling the input beam to specific cores through SLMs, as demonstrated in the aforementioned studies [a, b], precise control of the excited mode in the addressed core is achieved without the need for mechanical adjustments, allowing real-time reconfigurability of focus positions. This selective excitation mechanism allows dynamic and adaptive focus tuning, making the system highly suitable for applications requiring fast and remote focus control.

Complex beam generation: In addition, the ability of SLMs to manipulate the output beams from a multicore fiber significantly expands the scope of our approach. By adjusting the phase or amplitude of the light exiting each core, SLMs can add beam-shaping functionalities beyond the intrinsic hologram capabilities, providing finer control over focal spot size, shape, and distribution. For example, as shown in [link], the coherent combination of multiple tailored Gaussian beams emitted from the cores of an MCF amplifier enables the creation of complex beam types, including higher-order linearly polarized modes, cylindrical vector beams, and optical vortex beams with helical phase fronts. In this paper, each core acts as a light source with tailored beam properties, with the SLM controlling amplitude, polarization, and phase. This hybrid approach can extend the capabilities of the metafiber approach for sophisticated optical manipulation tasks such as custom light field shaping in microscopy, optical trapping, and telecommunications. In addition, the metafiber concept may also be used to reveal nonlinear pulse dynamics in MCF systems, as noted in the paper referenced by the Reviewer (e.g., [link]).

To respond to the Reviewer comment, the main text has been amended as follows: … In addition to the single-core excitation presented in this work, spatial light modulators (SLMs) or digital micromirror devices (DMDs) could potentially be used to excite mode in multiple cores simultaneously, providing an additional degree of freedom for research exploration. **This offers exciting possibilities for creating complex beams with tailored properties, as the SLM allows precise phase or amplitude control of the output light of each core. For example, as demonstrated in [link], the coherent combination of multiple tailored GBs from the cores of an MCF amplifier facilitates the creation of complex beam types. Here our concept could potentially integrate beam-combining optics directly onto the fiber end face, paving the way for an all-fiber device capable of producing higher-order linearly polarized modes, cylindrical vector beams, and optical vortex beams with helical phase fronts. The combination of the holographic lenses concept and MCFs may also be used to reveal nonlinear pulse dynamics in MCF systems (e.g., [link]). Moreover, integrating SLMs into our concept enhances the flexibility and functionality of our**

metafiber system by enabling precise core excitation without mechanical adjustments. …

R1.3: Please discuss in more detail the limitations of the technology, for example, single hologram provides fixed positions of the focal spots from individual core beams. May the proposed approach to be modified somehow to become reconfigurable that important for many applications.

We appreciate the Reviewer's insightful comment regarding the limitations and potential reconfigurability of our approach. While the holograms used here provide fixed focal positions for each core, we are actively exploring an approach in an ongoing research project focused on intensity responsive holograms. These holograms are designed to be sensitive to an electric field distribution such that a given field profile correlates with a given focal position in the image plane. In this soon-to-be submitted study (manuscript ready for submission), relative power-controlled spatial focus shifts are demonstrated within a fully fiber-integrated configuration using a 3D nanoprinted intensity profile sensitive hologram. While we do not wish to reveal further details in order to preserve the novelty of our forthcoming publication, another potential approach to achieving reconfigurability is to use SLMs to adjust the phase or amplitude of the light entering each core, potentially allowing for fine-tuning the output focal positions through phase-sensitive holograms. Although challenging, especially for on-fiber integration, these methods have significant potential for applications requiring flexible and adaptive focal control.

To address the Reviewer's comment, we have expanded the main text of the manuscript as follows, taking care not to disclose details that might compromise the novelty of our forthcoming study: … [*new text from R1.2*] **Moreover, integrating SLMs into our concept enhances the flexibility and functionality of our metafiber system by enabling precise core excitation without mechanical adjustments. Note that current investigations are aimed at developing methods for creating reconfigurable focal positions, including responsive holograms and other adaptive approaches, to increase flexibility in applications requiring dynamic focus control.** …

[a] D. Lin et al. Nat. Comm. 11(1), 1–9 (2020).
[b] D.S. Kharenko et al. Proc. SPIE, vol.12775: Quantum and Nonlinear Optics X, 127750W (2023) https://doi.org/10.1117/12.268854

Response to comments of Reviewer #2

In this paper, the authors present an approach for remote focus manipulation using 3D nanoprinted holograms integrated on the end face of multi-core single-mode fibers. This method enables precise focus control within a monolithic metafiber device by allowing light coupled into any of the 37 cores to be precisely focused at predefined locations. The authors mention this research marks the first successful use of multi-core fibers for remote focus control via 3D nanoprinting, achieving crosstalk-free operation at visible wavelengths. Key findings include strong agreement between design, simulation, and experimental results, highlighting the potential of this technology to improve applications in fields such as biological optics, laser micro-machining, telecommunications, and laser surgery. This article is clear, concise, and suitable for the journal's scope.

We thank the Reviewer for the thorough review and positive assessment of our manuscript. We appreciate the insightful comments and are committed to addressing each point raised. In the sections that follow, we provide a detailed, point-by-point response to each comment.

Several suggestions are supplied:

R2.1: Suggest the authors enhance the introduction part, such as the background also the motivation for this work.

We appreciate the Reviewer's comments about improving the introduction. As no specific issues were highlighted, we have made several changes to the Introduction and hope that these changes meet the Reviewer's expectations:

… Light focusing **at specific locations** is fundamental for nearly all optical systems and is critical in many scientific and application areas, including **fields as diverse as** telecommunications (e.g., wavelength multiplexing [REF]), medicine (e.g., laser ablation [REF], biophotonics (e.g., optogenetics [REFs]), and materials science (e.g., laser micromachining [REF]). Many applications require remote **and ideally reconfigurable** focusing of light, which is generally difficult to achieve with bulk optics. **With the ability to deliver light over long distances while maintaining high transmission quality, optical fibers have become indispensable in modern photonics due to excellent light transportation capabilities**. **Many practical fibers consist of a high refractive index core surrounded by a low refractive index glass cladding (usually silica), forming solid glass fibers with a step-index type profile.** These fibers have revolutionized photonics in various fields such as telecommunications [REF], quantum technologies [REF], and sensing [REF], thus suggesting a promising solution for remote light focusing when integrated with optical focusing capabilities. …

… One drawback of all mentioned on-fiber devices is that the generated focus remains static, while many applications demand real-time variable focus manipulation such as optical trapping and optical tweezers [REF], multiplexing in telecommunications [REF] or laser-based 3D nanoprinting [REF]. Here, reconfigurable optics is a promising field with the potential to transform technologies by enabling dynamic control of optical properties, increasing versatility and efficiency in applications such as imaging, sensing, and telecommunications [REF]. **From a technological standpoint, developing an integrated, fiber-coupled platform that reduces dependence on bulk optical components would decrease the system's geometric footprint and cost, creating new opportunities in reconfigurable optics.**
Multicore fibers with single-mode cores (SM-MCFs) are a topical research focus in fiber optics, overcoming key limitations of traditional fibers. One class of SM-MCFs is specifically designed to suppress modal crosstalk by confining power to individual cores [REF]. This improves system performance in several applications, including power scaling in fiber lasers [REFs], increasing data rates in telecommunications [REF], imaging complex tissues [REF], beam shaping [REF], and designing highly efficient sensors [REFs]. **Here, SM-MCFs provide a compelling platform for complex beam generation, as demonstrated by the coherent combination of multiple phase- and amplitude-tuned output beams from MCF amplifiers [link] and high-power laser applications [link], as well as for studying nonlinear pulse dynamics within MCF systems [link], [link].** Consequently, interfacing SM-MCFs with

phase-only holograms is expected to boost the performance of optical fibers and open up untapped applications, especially in tunable remote light focusing **and fields related**. …

R2.2: The authors use 37 GeO2-doped core, any specific reason?

We thank the Reviewer for the comment regarding the number of MCF cores used in this work. From our perspective, the use of 37 cores brings the following advantages:

> *Enhanced beam control and flexibility:* The use of 37 cores offers significant potential for beam and focus control, as opposed to a fiber with fewer cores, which would limit the number of foci that can be generated.
>
> *Optimal core density:* The hexagonal arrangement of the 37 cores maximizes core density while minimizing modal crosstalk (a separate study of the properties of this MCF is in preparation). A linear arrangement of the cores, for example, would accommodate far fewer cores, i.e. show a lower core density [link]. Note that in addition to the intercore pitch, a sufficient distance between the outermost cores and the cladding surface is necessary to prevent external influences to impact the light propagation inside the fiber.
>
> *Symmetrical core arrangement:* The hexagonal 37-core design creates a highly symmetrical arrangement of cores in the silica cladding, ensuring uniform core dimensions during fiber drawing and resulting in consistent beam parameters across all cores. In contrast, a linear core arrangement can result in uneven force distribution during fiber drawing, reducing the likelihood of maintaining identical core dimensions.

To account for the Reviewer's comment, we have modified the main text of the manuscript as follows: … In addition to the phase-only hologram, a key advantage of the concept presented here is SM-MCF that is single-mode at visible wavelength and thus is insensitive to bending. This is in stark contrast to systems using multimode fibers, where bending changes the transmission matrix, affecting the output beam profile and consequently focusing characteristics. In contrast, the SM-MCF concept is substantially less susceptible to bending, making it suitable for a wide range of applications. **The use of 37 cores greatly increases the flexibility of beam control, allowing a large number of foci to be generated that would be limited in case fewer cores are considered. In addition, the hexagonal core arrangement maximizes core density while maintaining negligible modal crosstalk and ensures uniform core dimensions during fiber drawing, unlike linear core arrangements that can lead to uneven force distribution and core variability during fiber implementation.** …

R2.3: The authors perform the 3D nanoprinting of on-fiber holograms using femtosecond direct laser writing, suggesting the authors enhance the achievement of this work.

We thank the Reviewer for the comment about improving the discussion of the results of our study. To address this comment, we have made the following changes to the text:

Implementation and experiments section: … 3D nanoprinting of on-fiber holograms was performed using femtosecond direct laser writing (fs-DLW) via two-photon absorption with a commercial photolithography system (Photonic Professional GT, Nanoscribe GmbH, details in Methods). **This technique offers several key advantages, including high spatial**

resolution and the ability to directly fabricate structures on unconventional surfaces like fiber end faces. Note that in contrast to wafer-based lithography, 3D nanoprinting is well suited for the fabrication of on-fiber nanostructures. The performance of the holograms is largely determined by the voxel dimension (300 nm transverse, 700 nm axial), which is a key factor in defining the spatial printing resolution. **Therefore, the fs-DLW process ensures a high degree of accuracy in replicating the computationally designed phase profile, making it possible to translate theoretical designs into practical, high-performance holographic devices.** To improve adhesion and prevent delamination, …

Discussion section: … In contrast, the SM-MCF concept is substantially less susceptible to bending, making it suitable for a wide range of applications. **The use of 37 cores greatly increases the flexibility of beam control, allowing a large number of foci to be generated that would be limited in case fewer cores are considered. In addition, the hexagonal core arrangement maximizes core density while maintaining negligible modal crosstalk and ensures uniform core dimensions during fiber drawing, unlike linear core arrangements that can lead to uneven force distribution and core variability during fiber implementation.**
The use of fs-DLW for on-fiber 3D nanoprinting is critical for this work, enabling the precise fabrication of on-fiber holograms with a level of detail and accuracy that directly translates into superior optical performance. This technique not only supports the implementation of complex holographic designs but also allows for future scalability to more intricate or application-specific hologram structures. The ability to fabricate such detailed nanostructures on small and unconventional surfaces such as a fiber end face showcases the potential of fs-DLW to advance integrated photonic devices and unlock new functionalities in optical systems. …

R2.4: Suggest the authors supply more detail in sentences about measured spatial intensity distribution (conf CF, linear scale) in the IP for modes excited in selected cores (core labels at the top right of each plot).

We thank the Reviewer for the comment to expand the discussion of the spatial intensity distribution of the conf_CF configuration. Since no specific aspect was mentioned by the Reviewer, we have expanded the discussion in the main text of the manuscript as follows: …
Experimentally, light was coupled into different cores of the hologram-enhanced SM-MCF and the resulting beam profiles were measured in the image plane (IP). Figures 3(a)-(i) show a selection of measured intensity distributions, with the corresponding cores noted in top-right of each figure. **Clearly visible in the images are locally concentrated light distributions, with the majority of the power localized at a specific location in the xy-plane, depending on the excited core. Note that outside the region of high intensity concentration, the power is significantly lower and distributed over larger spatial areas, allowing a single focus to be clearly assigned to each excited core.** The correlation between the excited core and the focus distribution is clearly visible in the arrangement of the measured focus positions. For example, excitation of the leftmost core (core C), the upper right core (core B), or the central core (core G) produces foci at R_C = (5µm, 0) (position "d", Fig. 3(h)), R_B = (-2.5µm,-5µm) (position "d", Fig. 3(i)), and R_G = (0,0) (position “g”, Fig. 3(a)), all of which correspond to the positions indicated in the pattern (red circles).
Interestingly, focal spots are also produced when intermediate cores, not included in the GSA design, are excited**, showing highly local intensity distributions and much lower power**

outside these regions. For example, Figs. 3(b), (e), and (h) show a discrete focus shift in the positive x-direction, suggesting that the design procedure effectively allows to create foci for all cores within a single IP. Most cores focus at the intended and expected positions (red and black circles in Fig. 3). Deviations may be due to printing imperfections or misalignment of the hologram with the fiber axis. …

R2.5: Suggest the authors supply more detail in sentences about the results of examples measured intensity distributions of the line-array focusing configuration.

We appreciate the Reviewer's suggestion to expand the discussion of the spatial intensity distribution of the line array configuration (configuration conf_LF). As no specific aspect was highlighted, we have modified the main text of the manuscript as follows: … Experimental characterization **relying on launching light into the six most outer cores and the central core (design cores)** using the same procedure as for previous configurations shows that single **spots of highly localized intensity** can be experimentally generated along a horizontal line (c.f. plots in the lower row of Fig. 9). **The corresponding positions match those defined by simulations and design (red and blue dashed circles), with a maximum shift of the focus from the center position of 29 µm. The intensity outside these areas of high field concentration is relatively low and unevenly distributed, indicating that a clear correlation between the addressed core and the location of the corresponding focus can be achieved.** Note that due to the complex correlation between the foci and their associated cores, slight deviations from the linear arrangement and a reduced symmetry of the foci intensity distributions are observed for the foci generated by off-axis cores (i.e. cores not aligned with the horizontal dashed lines in Fig. 9). …

To conclude, this work has merit and also has the potential to improve some applications.

Response to comments of Reviewer #3

The manuscript "Advanced remote focus control in multicore meta-fibers through 3D nanoprinted phase-only holograms" by Khosravi et al. reports on the fabrication of a phase-only hologram on the end facet of a multicore optical fiber that allows for remote focusing depending on the excited core.
The paper's concept is novel and clever; it will surely have a large impact. As such, it meets all the high-standard criteria for publication in Nature Communication.
The results are beautiful and interesting. By reading the manuscript, one may really feel the care taken to achieve the efficiency of the nanostructure fabrication process.

We thank the Reviewer for their thorough review and positive evaluation of our manuscript. We are grateful for the valuable feedback and have thoroughly responded to each point raised by the following point-to-point reply.

Still, before recommending the manuscript publication, I have a few comments that I would like to Authors to consider.

R3.1: The first question that came to my mind when reading the manuscript was "Why use 37 cores if, eventually, one only gets 7 focusing points?". I feel that this aspect is crucial and still

not discussed in the manuscript. Specifically, is a mismatch between the number of cores and the number of focusing points (i.e., holograms) needed, and why?

We thank the Reviewer for the valuable comment on the number of cores and foci. Here we would like to mention that the discrepancy mentioned by the Reviewer is indeed not clearly explained in the manuscript. In the following clarification, we provide a detailed explanation that has been added to the manuscript.

The multicore fiber used in this study has 37 cores in a hexagonal arrangement. Note that Reviewer #2 requested an explanation for the use of 37 cores in her/his second comment, which we explain in detail in the corresponding response (see R2.2).
The design procedure for all holograms implemented in this study (conf_CF, conf_SF, conf_LF) includes individual holograms from Gaussian field distributions generated by the six outermost cores and the central core, along with seven defined focal positions in the image plane. Note that these cores were chosen because they have the maximum possible spacing between the cores and thus the Gaussian beams in the hologram plane. Consequently, the final phase-corrected hologram is a superposition of seven individual holograms. The optical characterisation has shown that the different configurations behave in different ways:

> *conf_CF:* In the cross-axis focusing configuration, intensity measurements in the image planes revealed that in addition to the seven foci produced when launching light in the design cores, additional foci appear when the mode of any other core is excited, overall allowing to generate 37 position-controlled foci.
>
> *conf_SF and conf_LF:* For these configurations, localized intensity distributions that resemble a localized focal spot in the image plane appear only when light is launched into one of the design cores (six most outer and central cores). Introducing light into any of the other cores will result in uneven light distributions without a clear localized spot. Thus, for these two configurations, the multicore fiber with the 37 cores effectively functions as a 7-core fiber with an intercore spacing of 45 µm.

Therefore, while it would have been ideal to produce a new 7-core multicore fiber, we chose not to do so because this study serves as a proof-of-concept and the 37-core fiber can function effectively as a 7-core fiber. Consequently, we chose to use the existing 37-core fiber in these scenarios because creating a new fiber with similar functionality would have required additional time, manufacturing effort, and resources that would not have been an efficient use for this conceptual study.

When considering the Reviewer’s comment we realize that this important point was not addressed in the manuscript and we apologize for the oversight. We have now clarified this aspect in the manuscript to provide a more complete explanation of the choice of the 37-core fiber.

To address the Reviewer's comment, we have expanded the main text of the manuscript at the following locations:

Discussion of configuration *conf_SF*: … To evaluate the performance, laser light was selectively **coupled to the six most outer cores and the central core (design cores) and**

the resulting intensity distributions in the IP were retrieved (Fig. 7(a-d)). Each image shows a strong local concentration of intensity resembling a focal spot, while outside these regions, light is barely visible and unevenly distributed. Note that in this configuration, the 37-core fiber effectively operates as a 7-core MCF with an intercore pitch of 45 µm. We chose this approach, as producing a new 7-core fiber with identical functionality to the 37-core fiber solely operating with the design codes would require additional time, manufacturing effort, and resources. The design (red dashed circles), simulations (blue dashed circles), and experimental results show strong agreement, verifying the use of the design procedure and the quality of the implemented structures. Note that same-side focusing cannot be achieved with a conventional hyperbolic phase profile lens, demonstrating the relevance of using a holographic approach to achieve such functionality. …

Discussion of configuration *conf_LF*:
… Experimental characterization **relying on launching light into the six most outer cores and the central core (design cores)** using the same procedure as for previous configurations shows that …

… and a reduced symmetry of the foci intensity distributions are observed for the foci generated by off-axis cores (i.e. cores not aligned with the horizontal dashed lines in Fig. 9). **Similar to the previous configuration (conf_SF), the 37-core fiber functions as a 7-core MCF with an intercore pitch of 45 µm.** …

R3.2: Overall, I believe that the quality of the presentation can be improved. The labeling of the foci and the nanostructures' positions may be hard to follow if not strongly supported with clear figures. I noticed, for instance, that Fig. 2(a-b) and Fig. 6(a-b) are identical. This makes it hard to understand which cases they refer to. In my opinion, an extra panel showing connecting arrows between the nanostructures' positions with the foci positions will be beneficial to improve the presentation of the results.

We thank the Reviewer for highlighting the importance of clearly presenting the correlation between the considered cores and focal positions in the image plane. In response, we have color-coded the disks representing the cores (Figures 2(a), 6(a), and 8(a)) and the focal positions in the image plane (Figures 2(a), 6(a), and 8(a)) to ensure that linked pairs share the same color. Additionally, we have included arrows and labels to emphasize selected examples of these combinations (see revised figures below). We hope that these improvements, along with updated captions, will meet the Reviewer's expectations for clarity.

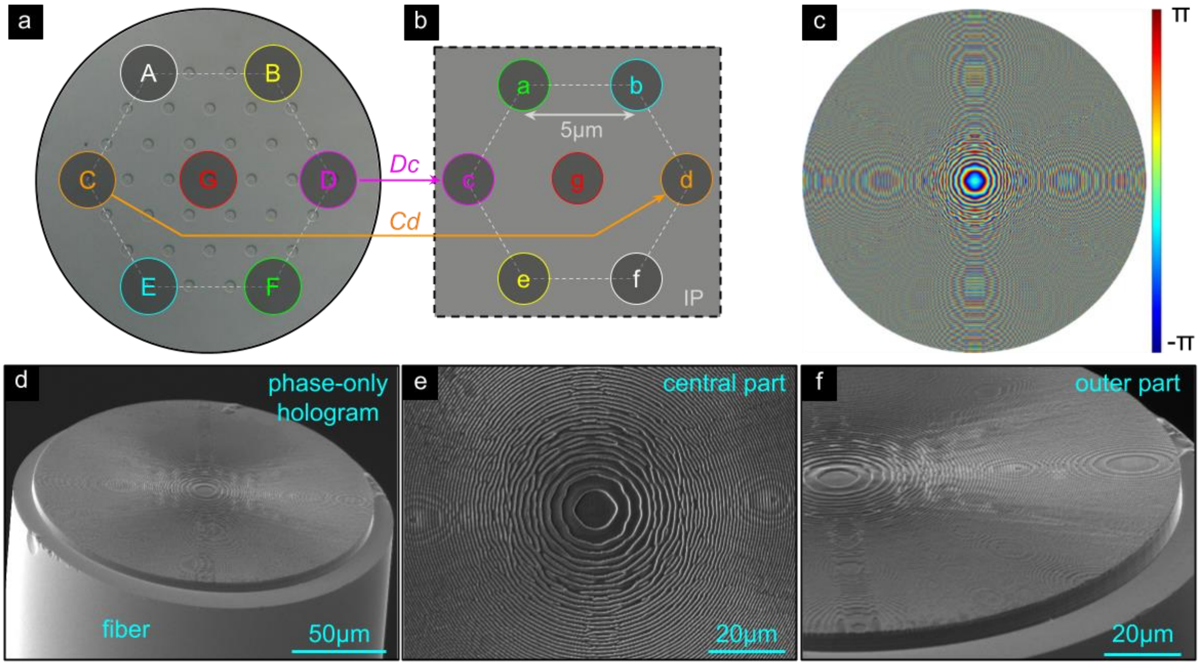

Fig. 2: Design and realization of the hologram-functionalized SM-MCF for cross-axis focusing (conf_CF, e.g. A -> f). (a) SEM image of the SM-MCF surface (cores have been etched for better visibility). The circles (cyan) indicate the positions of the cores used in the GSA-design procedure (core labels: A-G). The dashed line indicates the hexagonal lattice. (b) Hexagonal arrangement of the considered foci (purple circles) in the IP (foci labels: a-g). **To visualize the assignment of cores and focus positions (same color in (a) and (b)), two selected examples of core/focus position links are highlighted with arrows (orange: Cd, magenta: Dc).** …

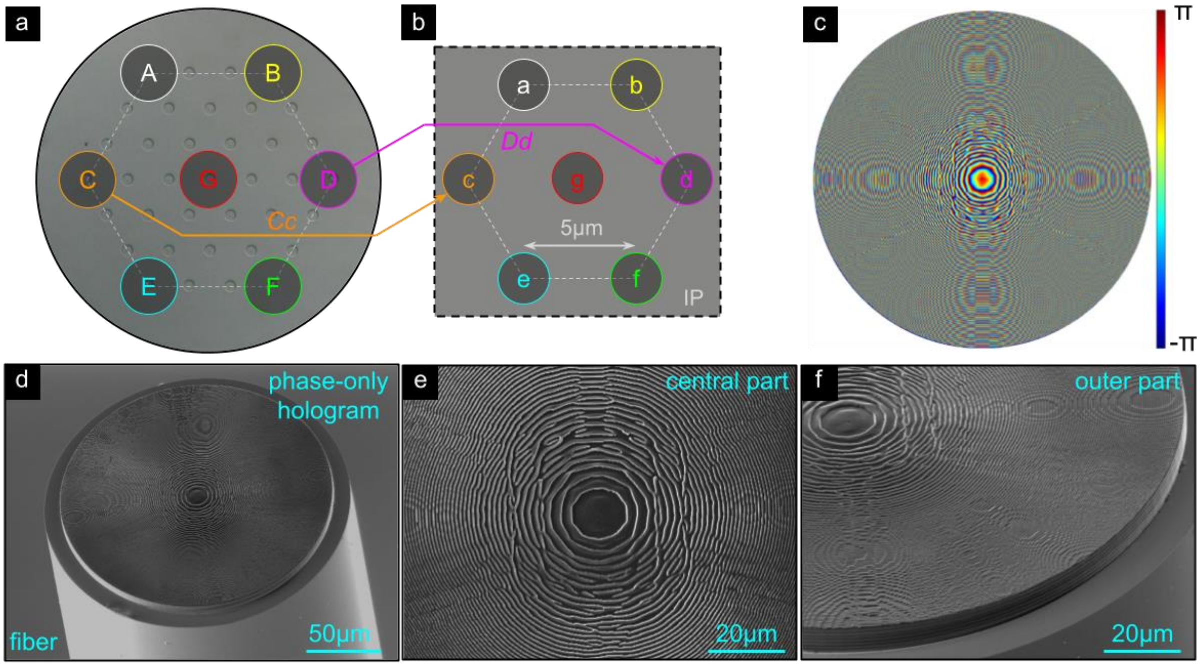

Fig. 6: Design and realization of the hologram-functionalized SM-MCF for same-side focusing (conf_SF, e.g. A->a). (a) SEM image of the SM-MCF surface, with cyan circles indicating the positions of the cores (core labels A-G). Dashed line: hexagonal

lattice. (b) Arrangement of the foci (purple circles) in the IP (foci labels: A-G). **To illustrate the assignments of core and focus positions (same colors in (a) and (b)), two examples are marked with arrows (orange for Cc, magenta for Dd).** …

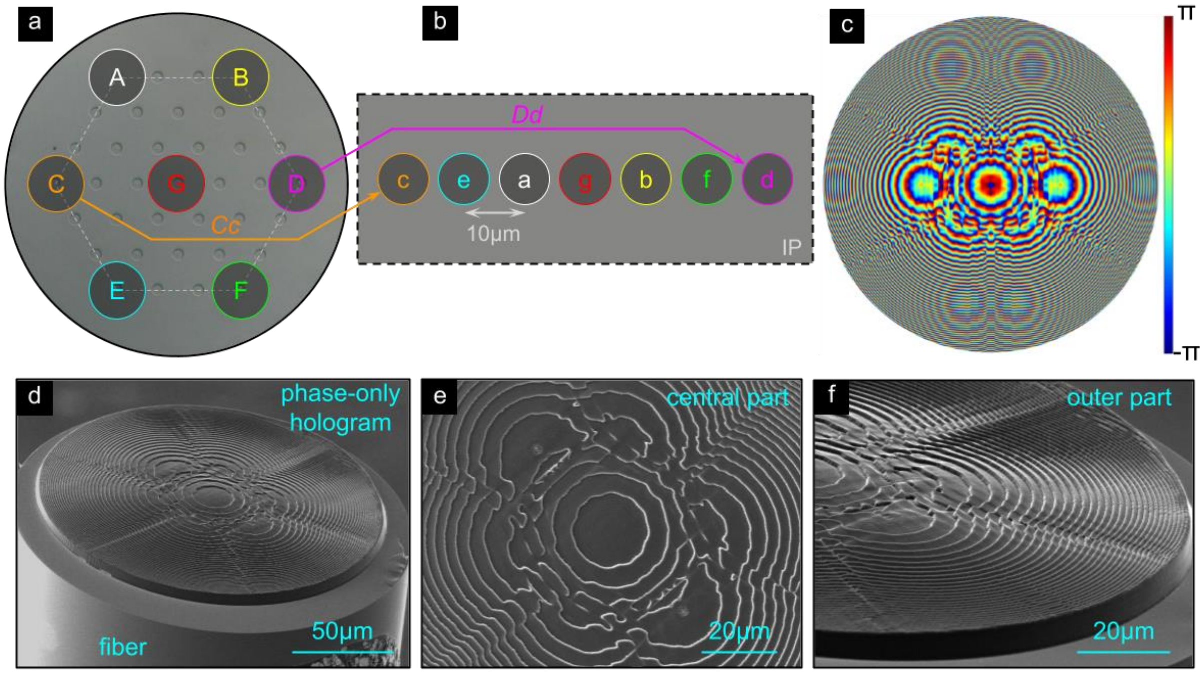

Fig. 8: Design and realization of the line-type focus arrangement (conf_LF). (a) Surface of the SM-MCF with circles (cyan) indicating the positions of the cores (A-G: core labels). (b) Line arrangement of the foci (purple circles) in the IP (A-G: focus labels). **Similar to the previous figures, linked cores and focus position share the same colors (two example assignments are highlighted by the arrows).** …

Minor points

R3.3: In line 31, a closing parenthesis ")" is missing after the word "ablation".

Thank you. Corrected.

R3.4: In line 34, the Authors specifically refer to step-index fibers. I believe that all types of optical fibers are efficient solutions for light transportation. No need to specify "step-index". In addition, in line 35, it is written that step-index fibers have doped core and undoped cladding. This is not always true. Often, indeed, step-index fibers are made of undoped silica core and Fluorine-doped silica cladding.

We thank the Reviewer for this comment, which we have addressed by including the following changes to the main text of the manuscript: … Many applications require remote focusing of light, which is generally difficult to achieve with bulk optics. **With the ability to deliver light over long distances while maintaining high transmission quality, optical fibers have become indispensable in modern photonics due to excellent light transportation capabilities. Many practical fibers consist of a high refractive index core surrounded by**

a low refractive index glass cladding (usually silica), forming solid glass fibers with a step-index type profile. These fibers have revolutionized photonics in various fields such as telecommunications [REF], quantum technologies [REF], and sensing [REF], thus suggesting a promising solution for remote light focusing when integrated with optical focusing capabilities. …

R3.5: In the introduction, there is quite an abrupt jump from line 38 to line 39. Perhaps, the Authors may consider sharpening it a little by saying something like “When it comes to the control of light focusing, a key area of photonic research is...”.

We thank the Reviewer for the comment, which we have addressed by the following text change: … **In the context of controlling the flow of light, a** key area of photonics research is dielectric nanostructures for complex beam manipulation [REF], primarily focusing on metasurfaces (MSs) and phase-only holograms. …

R3.6: The sentence “As a result, by exciting the mode of a specific core, the focus position can be dynamically controlled within the IP.” in line 94 is not clear to me. The expression “the mode of a specific core” is confusing since each core supports only one mode. Moreover, I do not understand how the dynamic control of the focus position results from the incorporation of the coreless fiber section.

We appreciate the Reviewer's comment and apologize for any confusion caused by the wording of this sentence. To clarify, our intent was to convey that by selectively exciting different cores in the MCF, we achieve dynamic control over the focus position in the image plane (IP). While each core supports a single mode, the position of each core within the hexagonal array correlates to a unique focal spot position in the IP. By incorporating a coreless fiber section, the emitted beams from each core undergo controlled diffraction, which expands the beam area and matches the spatial extent of the hologram, improving the quality and precision of each focal spot. This setup allows the position of the focal spot in the IP to shift depending on the specific core being excited, providing dynamic focus control.

In light of the Reviewer's comments, we have revised the sentence for clarity in the main text of the manuscript as follows: … The concept of remote focus manipulation involves the use of SM-MCFs in combination with computer-designed, nanostructured phase-only holograms. This arrangement links each fundamental mode in the different cores with a focus position in the image plane (IP, located at $z = f$, f: focal length). **This design allows dynamic control of the focus position in the IP by selectively exciting a different core.** The SM-MCFs contain $N_c = 37$ cores and are designed to have no intermodal crosstalk in order to ensure a correlation between the light entering and exiting each core. To increase the efficiency of the nanostructured hologram, **and to fully utilize the hologram area, a coreless fiber section is added between the fiber surface and the hologram, allowing the beam from each core to expand via diffraction.** ~~As a result, by exciting the mode of a specific core, the focus position can be dynamically controlled within the IP.~~ …

R3.7: In line 115, it is specified, for the first time, that the nanostructure is made of a polymer material. I believe that this should be stated above.

We thank the Reviewer for this comment. We have now included this in the Introduction section: … In this study, we address the aforementioned aspect by introducing and experimentally validating a novel approach for remote focus manipulation by integrating computationally designed 3D nanoprinted **polymeric** holograms onto the end face of single-mode multicore fibers (Fig. 1). …

R3.8: The acronym "GB" is defined twice.

Thank you. We have changed the main text of the manuscript as follows: … The resulting hologram (Fig. 8(c)), designed by the ASM-GSA approach, is more complex compared to the previous designs (Fig. 2(c) and Fig. 6(c)) and shows indications of the ~~Gaussian beams (~~GBs~~)~~ in the phase distribution. …
